# Modelling of the Flow in the Process of Washing Out Automotive Catalyst Carriers with the PbLi Alloy

**DOI:** 10.3390/ma15093119

**Published:** 2022-04-26

**Authors:** Mariola Saternus, Agnieszka Fornalczyk, Władysław Gąsior, Adam Dębski, Sylwia Terlicka, Sławomir Golak, Piotr Ciepliński

**Affiliations:** 1 Faculty of Materials Engineering, Silesian University of Technology, ul. Krasińskiego 8, 40-019 Katowice, Poland; agnieszka.fornalczyk@polsl.pl (A.F.); slawomir.golak@polsl.pl (S.G.); piotr.cieplinski@polsl.pl (P.C.); 2 Institute of Metallurgy and Materials Science of the Polish Academy of Sciences, ul. Reymonta 25, 30-059 Kraków, Poland; w.gasior@imim.pl (W.G.); a.debski@imim.pl (A.D.); s.terlicka@imim.pl (S.T.)

**Keywords:** wettability, PbLi, catalytic converters, platinum recovery

## Abstract

The process of platinum recovery from used car catalysts is highly desirable for both economic and environmental reasons. From the many available methods of processing used car catalysts, the article conducted both numerical and experimental studies using a device based on the collector metal method with lead as a modified medium through a magnetohydrodynamic pump for washing platinum from the channels of the ceramic catalyst carrier. It was assumed that lead alloys with the addition of lithium increase the extraction of platinum from thin catalytic layers and accelerate the platinum dissolution reaction in the Pb-Li alloy, which is the result of a greater affinity of lithium for platinum compared to lead. This assumption was verified by numerical simulations as well as laboratory tests. Tests were carried out for the secondary supply voltage range between 40 and 60 V and the catalyst flushing time between 240 and 480 s. Graphical results of the research were discussed.

## 1. Introduction

Used car catalysts are currently a very important source of platinum group metals. Due to the production volume of cars and the increased environmental standards related to emissions, the demand for platinum group metals (Pt, Pd, and Rh) as a catalytic substance increases every year [1,2]. Currently, to keep up with the supply of platinum group metals, they are recovered through recycling, including used car catalysts. The associated benefits include not only economic benefits but above all environmental benefits: reducing the amount of landfilled waste, savings in the use of natural resources, or reducing energy consumption and emissions of pollutants from the technology of obtaining metals from ores [3,4]. Many methods of processing used car catalysts have been developed [5,6,7,8,9,10,11,12,13,14,15,16,17,18,19,20,21,22,23] applying the hydrometallurgical processes with the use of aqua, cyanides, chlorine, chlorides, chlorates, nitrates, or bromates [5,6,7,8,9,10,11,12,13] and pyrometallurgical processes with the use of other metals (e.g., iron, lead, copper, nickel) or materials (used printed circuit boards) constituting a liquid matrix for the accumulation of platinum metals [14,15,16,17,18,19,20,21,22,23]. The metal collector method has many advantages, such as no formation of numerous waste solutions very aggressive to the environment, easy separation of slag from an alloy of metals and platinum metals, and a small number of chemical reagents [24,25]. An interesting solution is a simple and comprehensive technology of platinum recovery from used car catalysts, based on a modified collector metal method [26]. Liquid lead was used as the metal due to its relatively low melting point. The liquid metal is placed in an annular channel in a closed circuit into which the catalysts are immersed. Lead is propelled by a rotating electromagnetic field. It is the only device that uses magneto-hydrodynamic phenomena to leach platinum and other platinum group metals from catalytic carriers [27] (discussed in more detail in Section 3).

Lead alloys with the addition of lithium may increase the extraction of platinum from thin catalytic layers as a result of reducing the surface tension of the extraction liquid (Pb-Li), improving its penetration deep into the capillaries with catalytic layers and accelerating the dissolution reaction of platinum and palladium in the Pb-Li resulting from the greater affinity of lithium to Pt compared to lead. As part of experimental studies [28,29,30], the thermodynamics of lithium systems were determined (two- and three-component lithium-based alloys: Pd-Li, Pt-Li, Pt-Pb-Li, and Pd-Pb-Li). The next step is to determine if there is a clear influence of the lithium concentration in the Pb-Li alloy on the size of the extracted platinum and palladium from catalytic thin films in capillaries compared to pure lead. Research of this type, supported by numerical simulations concerning the intensity of magnetohydrodynamic flushing of catalysts, will be presented in the article.

## 2. Numerical Model of the Process

In order to start the laboratory tests, the device operating parameters should be selected. For this purpose, a virtual, numerical model of the process was built. The model considers the magnetohydrodynamic phenomena which are the driving force of the discussed process [31].

This required an analysis of issues related to the calculations of the electromagnetic field and the flow field. In mathematical modelling, the analysis of the electromagnetic field was based on the differential equation defining the distribution of the vector magnetic potential:

(1) $$\nabla \times \left(\frac{1}{\mu_{p}}\nabla \times \underset{-}{A}\right)+j\omega \sigma \underset{-}{A}=\underset{-}{J_{s}},$$

where *μ_p_* is the magnetic permeability, $\underset{-}{A}$ is the magnetic vector potential, j is the imaginary unit, *ω* is the angular frequency, *σ* is the electric conductivity, and J_s_ is the source current. The symbol “X” stands for complex values.

Once the magnetic vector potential equation was solved, it was possible to determine the magnetic induction using the following equation:

(2) $$\underset{-}{B}=\nabla \times \underset{-}{A},$$

where $\underset{-}{B}$ is the magnetic induction.

The final formulation allowed the calculation of the eddy current density and applied information regarding the magnetic vector potential from (1):

(3) $$\underset{-}{J}=-j\omega \sigma \underset{-}{A},$$

The results from the electromagnetic analysis were transferred into the fluid dynamics model. The Lorentz force acting on liquid metal was computed according to the formula:

(4) fm=12Re(J-×B-*),

where **f**_**m**_ is the electromagnetic force and ${\underset{-}{B}}^{*}$ is the complex conjugate of **B**.

In the conducted analysis, it was assumed that the amount of heat released as a result of electromagnetic interactions was negligible and that the entire system had to be resistively heated, and the resistive heating system allowed to maintain the system in thermal equilibrium. For the flow field, the design area is limited to the liquid alloy. In the free zone of the channel and the area of the porous, anisotropic catalyst structure, the flow field was modelled using the Navier-Stokes equation:

(5) $$\rho \left(\frac{\partial v}{\partial t}+v\cdot \nabla v\right)=-\nabla p+\eta_{e}\nabla^{2}v+f_{m},$$

where *η_e_*—effective viscosity is determined from the k-ε turbulence model.

In this area, a standard k-ε turbulence model was adopted. The capillary structure of the catalyst carriers requires a significant modification of the model representing the channel zone outside the catalyst carriers. 

The areas occupied by the catalyst carriers were modelled as an anisotropic porous medium with laminar flow and pressure drop, according to the Hagen-Poiseuille equation:

(6) $$\frac{\Delta p}{L}=\frac{28.5}{d^{2}}\eta_{d}v_{c},$$

where: Δ*p*—pressure drop along the length *L* of the capillary, *η_d_*—dynamic viscosity, *v_c_—*component of velocity parallel to the capillary.

In the catalyst area in the Navier-Stokes equation, the modulus representing the resistance of the porous medium appears. Since the macroscopic conductivity of this region is 0, no Lorenz forces are acting on the metal in this region. Therefore, the last source term in Equation (5) disappears:

(7) $$\rho_{f}\left(\frac{\partial v}{\partial t}+v\cdot \nabla v\right)=-\nabla p+\eta \nabla^{2}v-\frac{28,5}{d^{2}}\eta \left(n\cdot v\right)n-\infty \left(m\cdot v\right)m,$$

where: **n**—unit vector parallel to the direction of the capillaries, **m**—unit vector perpendicular to the direction of the capillaries, ∞—the largest possible number limited by the stability of calculations.

In the simulations, to enable numerical implementation, in the last module representing the resistance of the capillary structure in the direction perpendicular to the direction of the channels, instead of the value ∞, a number of several orders of magnitude greater than the resistance of the structure in the direction of the channels are inserted.

At the same time, the presence of the walls of the channels separating individual fluid streams translates into the lack of the moment of the module directly expressing the internal friction of the liquid in the equation. The internal friction was considered indirectly in the module derived from the Hagen-Poiseuille equation.

**Figure 1:**
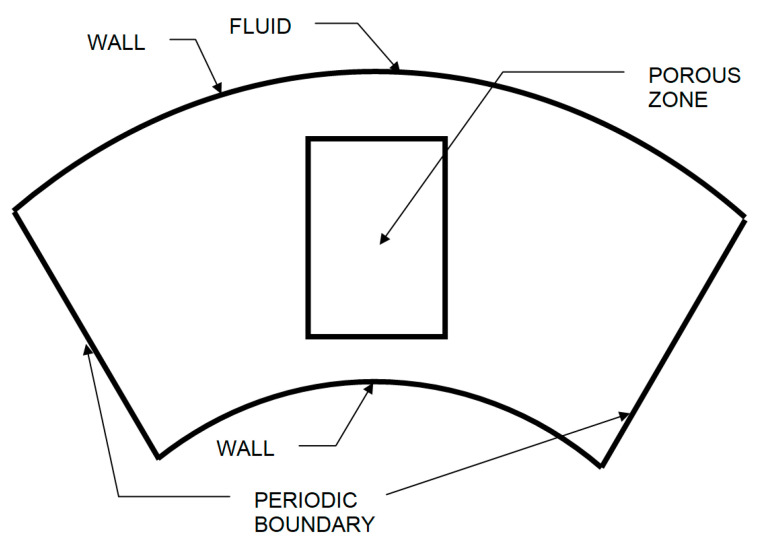
System geometry for the device channel model.

Using the developed numerical modelling methodology and based on the adopted assumptions, a series of numerical experiments for the model was carried out. The model was implemented on the basis of Ansys software, completed with its own code that realizes the coupling between hydrodynamic and electromagnetic fields. Figure 1 shows the geometry of the modelled system for the main part of the device—the channel with liquid metal and catalysts. The wall boundary condition was defined as no slip. The porous zone represents the capillary structure of the catalyst.

## 3. Materials and Methods

In order to enable the achievement of sufficiently high levels of extraction of platinum and palladium, tests and simulations were carried out to improve the wetting of the capillaries of the catalytic carriers by the addition of surface-active substances. One alternative in this regard may be the addition of lithium to the lead collector metal. Lithium has a low atomic number, a low melting point of only 179 °C, a high boiling point, and high surface tension [32,33]. Several plasma-facing components systems use liquid lithium, such as capillary porous systems (CPS) [34,35], in which wetting of the substrate with lithium is an important criterion [36]. Lithium must wet the surfaces of channels; what is more, if lithium is used in upside-down or vertical elements, it must also wet their surfaces [34]. Wetting of lithium compounds by liquid lithium was studied at various temperatures [32,37] and as a result, it was found that the presence of some lithium compound contaminants on the surfaces should not significantly hinder lithium’s ability to wet them and may even promote wetting. The spreading of molten metals can be decreased by decreasing interior atomic interactions by introducing elemental additives to form alloys or compounds. Because the bonding force between the heteroatoms and Pb is smaller than that of Pb–Pb, introducing a trace amount of elemental additives to pure metals to form alloys or compounds can reduce the interior atomic interactions to decrease the surface tension [38,39].

**Figure 2:**
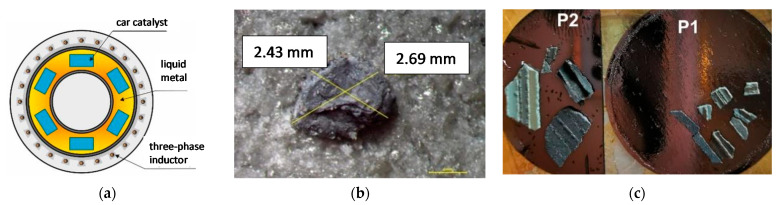
(**a**) Diagram of the device used in the experimental tests, (**b**) results of the PbLi droplet spread on the platinum plate, (**c**) prepared samples (placed on a graphite disc and sputtered with gold) for examination on a scanning microscope.

Experimental tests were carried out for used car catalysts from passenger cars. The content of Pt in the catalyst carriers and after the process was determined by inductively coupled plasma mass spectrometry (ICP-MS) with the PerkinElmer NexION 300D spectrometer. The determination was performed for samples prepared by the method of melting with sodium peroxide and dissolving the alloy in aqua regia (CRM—used ERM-EB504 car catalyst). The expected and desired effect of the experiments was the reduction of the Pt content in the ceramic carriers of the catalyst per unit time (due to a large amount of liquid metal Pb-Li and the low initial content of noble metals in the catalysts, it is not possible to determine the increase in their concentration in the liquid metal). Ceramic carriers of used car catalysts, after appropriate cutting, were placed in the device channel (Figure 2a), in which there was a liquid lead-lithium alloy. In this device, the recovery of platinum from used car catalysts takes place by washing it from the capillaries of the carriers thanks to the magnetohydrodynamic effect. The preliminary tests of the spread of the lead-lithium alloy (4, 8, 12, and 16 wt.%) on the platinum plate showed that the best results were obtained for the Pb + 4 wt.% Li alloy (Figure 2b) [27]. On this basis, an alloy with such a lithium concentration was used in the experimental studies. 

Before starting the research, the surface of the catalytic carriers was also tested for the degree of adsorption. Examination of the sample surface morphology was performed using the Hitachi SEM SU3500 scanning electron microscope. Before the measurement, the sample was dried in a laboratory dryer (50 °C). The test sample was placed on a graphite disc and dusted with gold (Figure 2c). This is a typical preparation used to obtain images using a secondary electron signal, in the case of non-conductive materials (made of ceramic materials). Gold provides the best surface representation with a relatively low material layer thickness.

The actual tests aimed at verifying the numerical simulations carried out were conducted with the use of a proprietary device—a magnetohydrodynamic pump for washing out used car catalysts with liquid metal (in this case Pb-Li). The operating temperature was 400 K. In the course of the experiments, an inert atmosphere was used to avoid the formation of oxides on the surface of the liquid lead. Tests were carried out for the secondary supply voltage range between 40 and 60 V and the catalyst flushing time between 240 and 480 s. Table 1 additionally presents the geometry parameters and material properties for a device for the recovery of platinum from spent catalysts using magnetohydrodynamics.

## 4. Results

### 4.1. Computer Simulation Results

Based on the developed mathematical model of the process, an analysis of magnetohydrodynamic phenomena occurring during the analyzed process of washing out the catalyst inserts was carried out.

**Figure 3:**
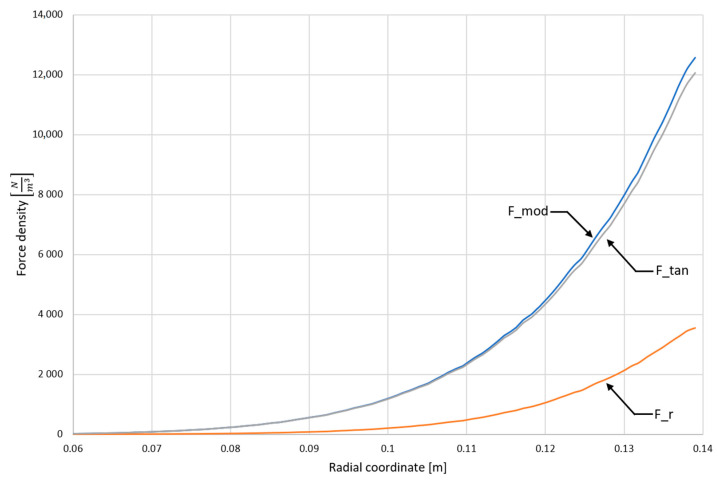
Electromagnetic forces act on the liquid metal in the channel from the supply current (voltage 60 V). F_r—force component perpendicular to the channel wall, F_tan—force component parallel, tangent to the channel wall, F_mod—EM force modulus—sqrt (F_r^2^ + F_tan^2^).

Figure 3 shows the distribution of electromagnetic forces acting on the liquid metal, measured across the channel for the 60 V supply voltage of the inductor in the area without the presence of the catalyst. This area is fully conductive and is the area of forced metal flow in the channel of the device. The greatest values of forces occur near the outer wall of the channel. The reason is the disappearance of the electromagnetic field generated by the inductor of the device as a result of its shielding by liquid metal. This distribution will be reflected in the distribution of the hydrodynamic field of the liquid metal.

**Figure 4:**
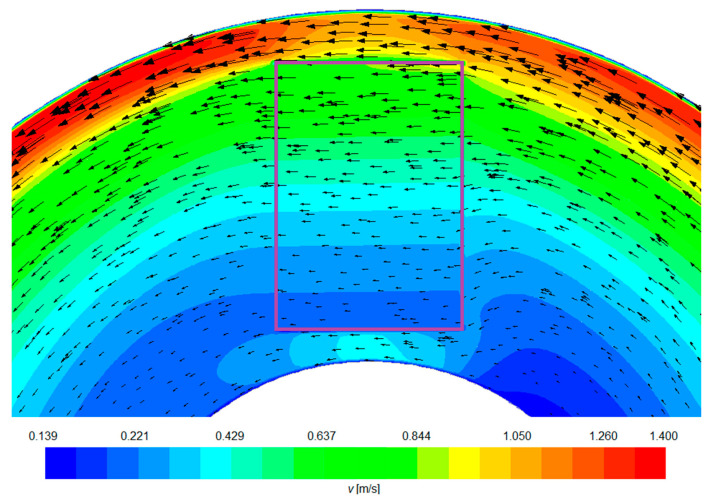
Catalyst flow field distribution for 60 V inductor voltage.

This is seen in Figure 4 showing the velocity field of the liquid metal in the region of the catalyst (red rectangle) and beyond. The greatest intensity of the flow is visible at the outer wall of the channel

Additionally, it can be observed that the catalyst channels force the metal to flow in parallel through its area. You can also see that the catalyst is blocking the flow, and the area closest to the axis is the worst flushed. The rate of infiltration of the catalytic carrier capillary structures by the liquid Pb-Li alloy can be increased by rotating the catalysts in an axis parallel to the liquid melt flow. However, this is a time-consuming solution that makes it difficult to automate the solution for commercial applications in the future. 

**Figure 5:**
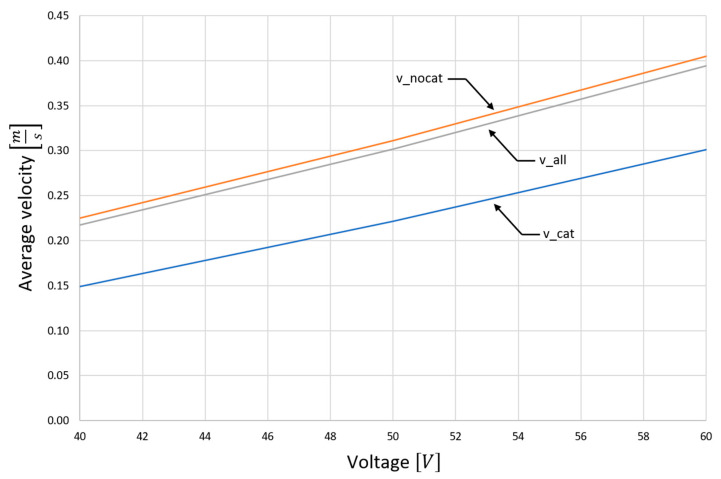
Average flow velocities in the device as a function of the secondary voltage of the inductor: v_cat—average flow velocity through the catalyst itself, v_nocat—apart from the catalyst, v_all—in the entire device.

Figure 5 shows the measures of average flow velocities in the channel for various exciter supply voltages, which determine the flow rate of liquid metal in the device. As can be seen, these parameters strongly depend on the analyzed area of the channel. For the porous structure of the flow-inhibiting catalyst carrier, the velocity is reduced by approximately 30%. 

**Figure 6:**
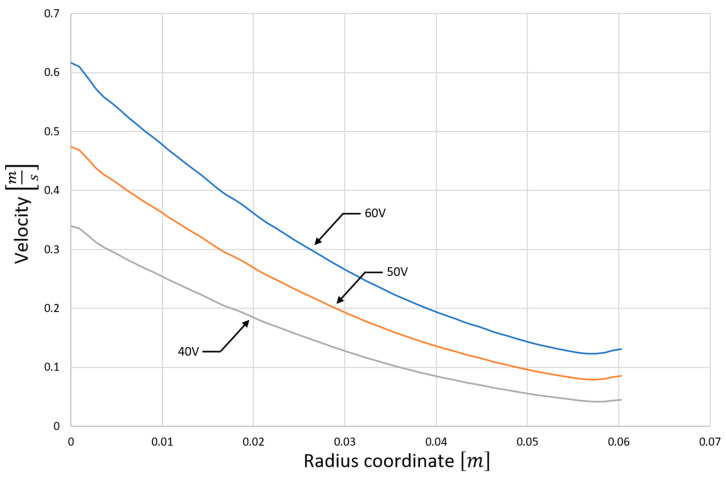
The flow velocity distribution in the catalyst (in the direction from the axis to the outside of the device) for the supply current of 40 V, 50 V, and 60 V.

Figure 6 shows the exact flow velocity distribution along the catalyst channels (for different supply voltages). One can notice the aforementioned significant difference in the flushing intensity of the various parts of the catalyst carrier. Obviously, increasing the supply voltage increases this intensity, but has a relatively small effect on the flow through the catalyst region near the axis of the device. 

**Figure 7:**
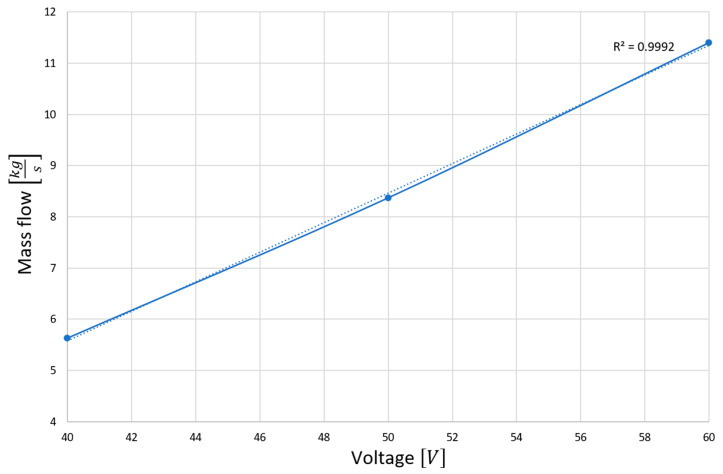
The amount of metal flowing through the catalyst per unit time as a function of the supply voltage.

The diagram presented in Figure 7 shows the dependence of the liquid metal flux in the function of the secondary voltage of the current supplying the inductor windings of the electromagnetic stirrer. In the analyzed range, an almost linear dependence of the amount of flushing metal on the voltage can be seen, which is confirmed by the coefficient of determination close to one (0.9992 for the linear relationship).

### 4.2. Analysis of Platinum Washing Out Results in a Device with a Magnetohydrodynamic Pump

**Figure 8:**
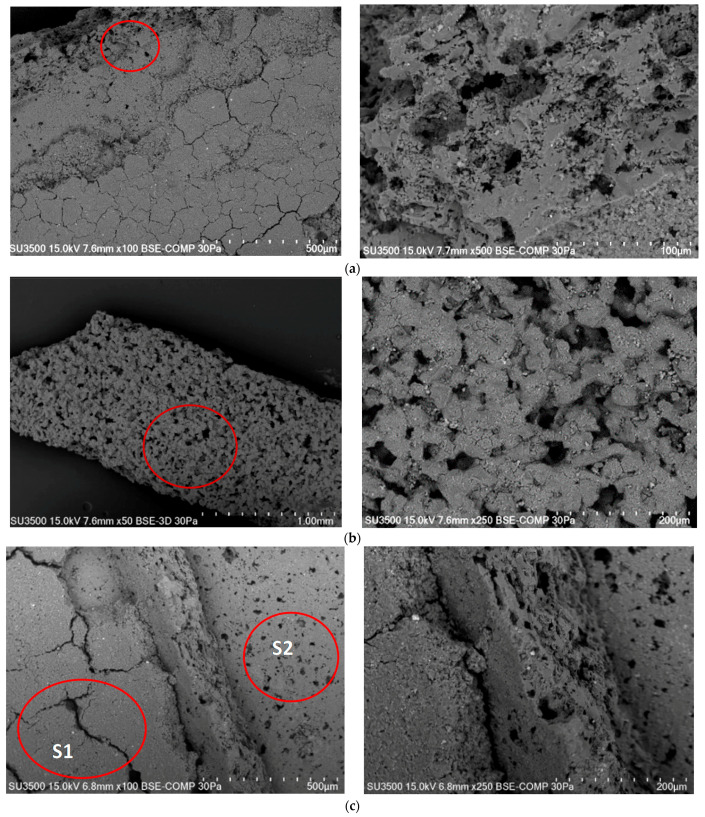

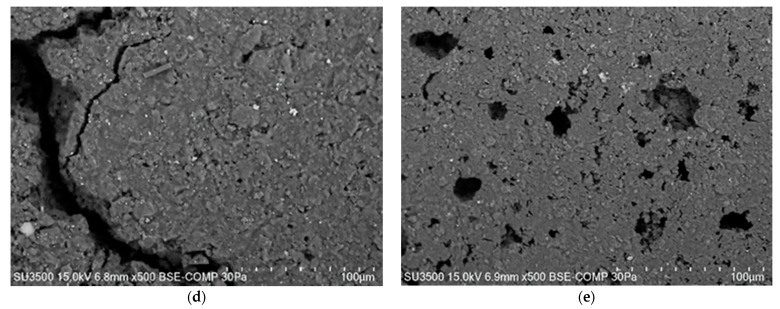
The surface structure of the catalytic carrier sample at selected magnifications for the sample: (**a**) P1—fragment 1, (**b**) P1—fragment 2, (**c**) P2, (**d**) fragment S1 from (**c**), (**e**) fragment S2 from (**c**).

Figure 8 shows the results of the catalytic carrier surface tests, Figure 8a shows a fragment of the P1 sample, and Figure 8b shows a fragment of the P2 sample at different magnification. Additionally, in the P2 sample in two different places, it was decided to take pictures of the structure under magnification. At site S1, a crack and a large crevice were observed, which in turn will increase the degree of surface adsorption. At the S2 site, you can see fine pores that, when enlarged, turned out to be irregular defects in the surface of the carrier. Based on the results obtained, it can be concluded that the material is porous, and gaps are observed in it. This indicates that the catalytic carrier is well wettable by substances such as lead, of course, the addition of lithium should increase this degree of wettability.

**Figure 9:**
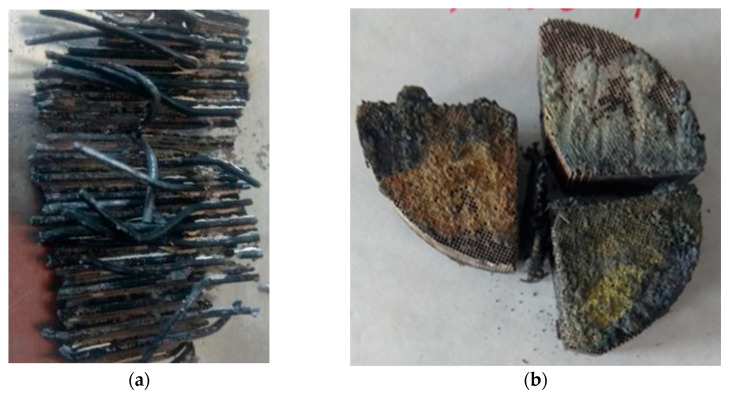
Catalyst cross-section along the liquid metal flow axis with visible alloy rods (**a**); the front surface of catalyst carrier with remains of metal at outlets of capillaries (**b**).

Taking molten metal flow measurements is no easy task. A number of measurement methods have been developed [25], but none of them is suitable for measuring the flow of metal through the porous structure of the catalyst. A simple way to check the reliability of the presented infiltration model is to place the catalysts in the liquid metal stream, remove them after infiltration and check which capillaries have been filled with the molten metal. The problem is that after removing the catalyst from the channel, liquid metal still flows from the carriers’ capillaries under the influence of gravity and capillary pressure. For this reason, the efficiency of capillaries infiltration can be assessed by verifying the amount of solidified metal at its outlet (Figure 9). Based on the above, it can be concluded that all pieces of catalyst carriers (capillaries) have been wetted with liquid metal.

An indirect method of verifying numerical calculations is the measurement of the loss of platinum in the catalyst as a function of the parameters of the current feeding the electromagnetic stirrer. The method has the additional advantage that it represents the real, practical goal of the analyzed process.

**Figure 10:**
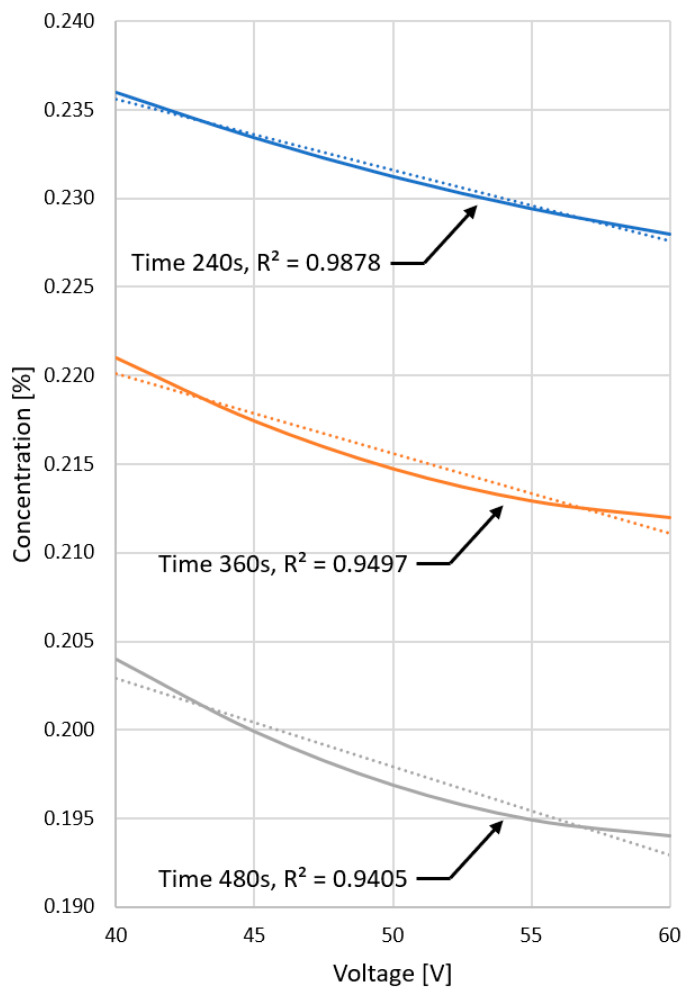
Graph of the dependence of the change in Pt concentration in automotive catalysts as a function of the secondary voltage for various flushing times.

The graph in Figure 10 shows the dependence of the platinum loss as a function of the supply voltage for different washing-out times of the catalyst carriers. For the time of the 240 s, when the amount of platinum in the catalyst structure remains high throughout the washing-out period, a linear dependence on the supply voltage of the inductor can be observed. It is related to the linear dependence of the intensity of washing out the catalyst carrier on the supply voltage (determined based on the numerical model of the process), as shown in the diagram in Figure 7.

As soon as the catalyst is running low on platinum, the rate of reduction of the platinum concentration decreases. For short flushing times, the concentration drop is linearly dependent on the supply voltage (flushing intensity) since platinum is available at all times. With longer times, platinum is depleted, and the decrease is no longer linear as a function of the flushing intensity. With longer flushing times, the effect of the loss (the disappearance of platinum in the catalyst) becomes more and more apparent. You can see the effect of the lack of platinum faster (for lower flushing intensities).

## 5. Conclusions

The process of washing out catalyst carriers with liquid alloy is a complex process in which there are many interacting physical phenomena. The main driving mechanism of the process is magnetohydrodynamic phenomena—that is, the coupling of electromagnetic and hydrodynamic fields.

The complexity of the process and difficulties with measuring the flow of liquid metal require tools that allow us to understand this technological process and select its control parameters—represented primarily by the supply voltage of the electromagnetic stirrer inductor.

The conducted research based on numerical simulations allowed for an effective analysis of the process, explaining its mechanism and the influence of power supply parameters on its course.

Based on the conducted laboratory experiments with the use of a device with a magnetohydrodynamic pump with selected process parameters, it can be concluded that lead alloys with lithium addition increase the extraction of platinum from thin catalytic layers as a result of reducing the surface tension of the extraction liquid (Pb-Li) and improving its penetration into the capillaries with catalytic layers and acceleration of the platinum dissolution reaction in the Pb-Li alloy, which is the result of a greater affinity of lithium for platinum compared to lead.

## Figures and Tables

**Table 1:** Geometry and material properties of the device and materials used in the experiments.

Parameter	Value
The outer radius of the channel	0.139 m
The inner radius of the channel	0.61 m
The inner radius of the inductor	0.149 m
The frequency of the power supply to the inductor	50 Hz
Pb-Li alloy density	10,298 kg·m^−3^
Lead viscosity with lithium	0.002 Pa·s
PbLi electrical resistivity	10,016,106 Ωm
Carrier width	40 mm
Carrier height	60 mm
Medium hydraulic capillaries	0.00085 m

## Data Availability

Not applicable.
